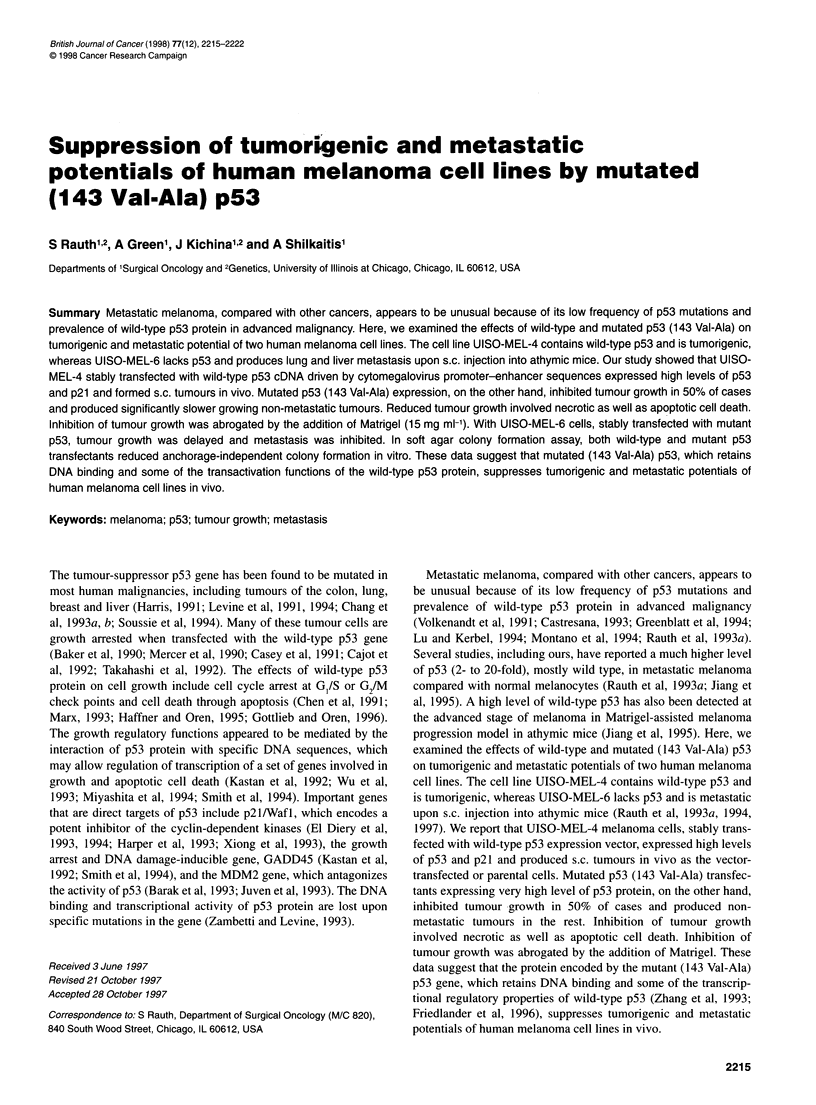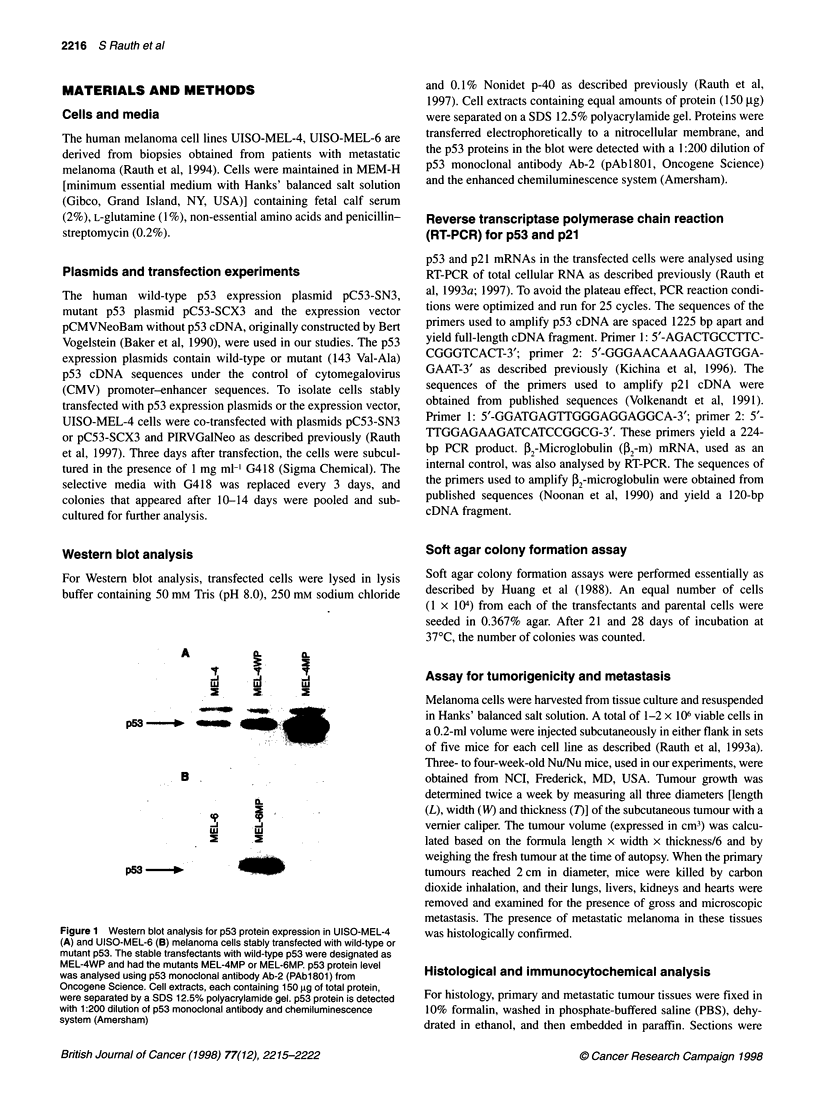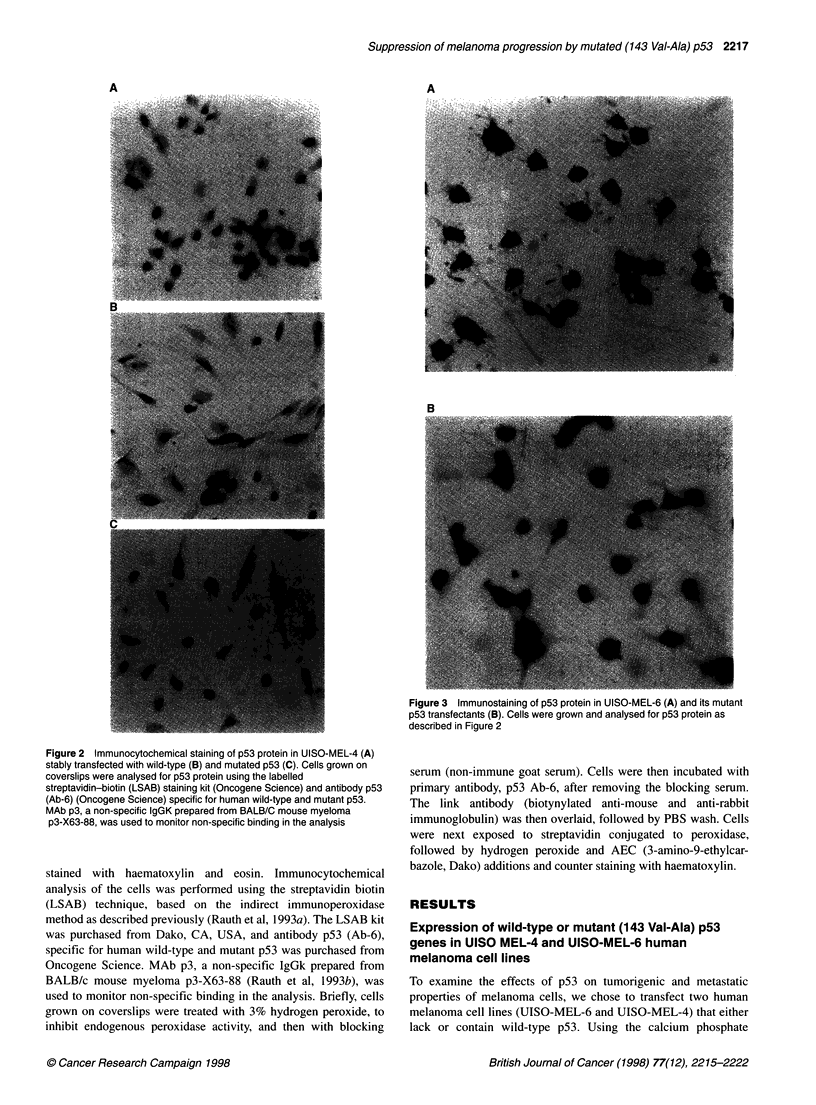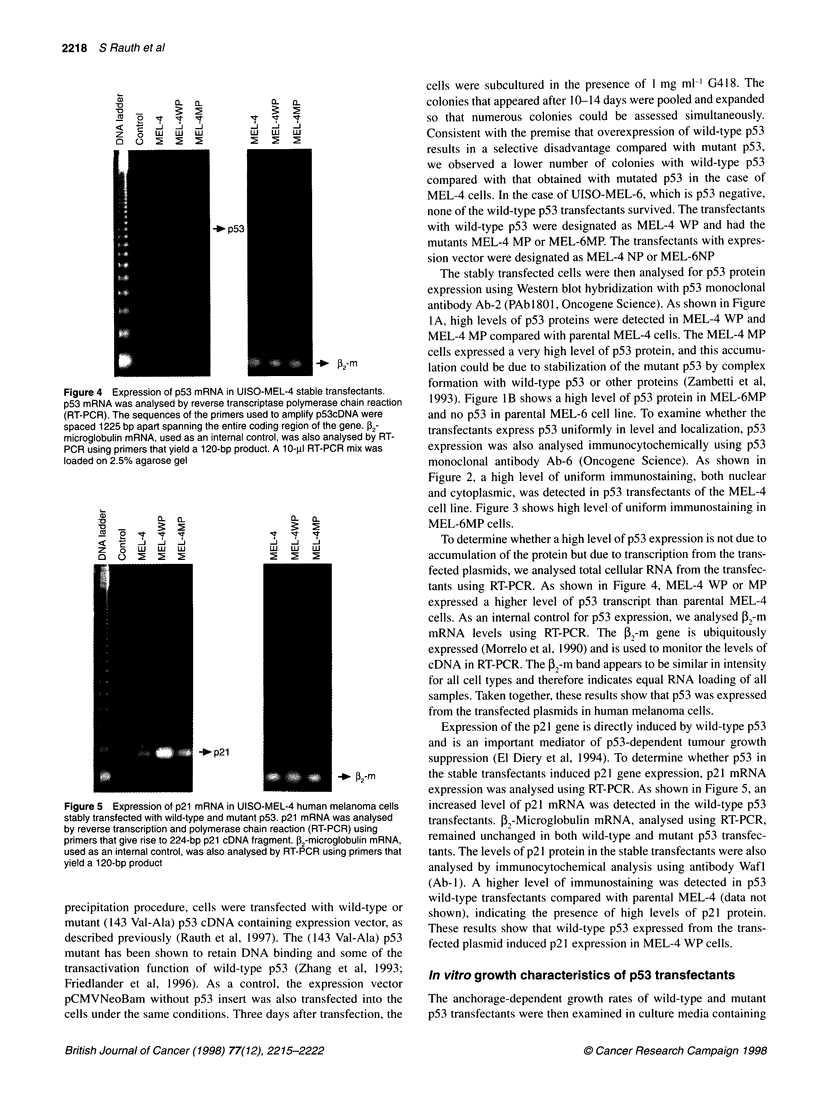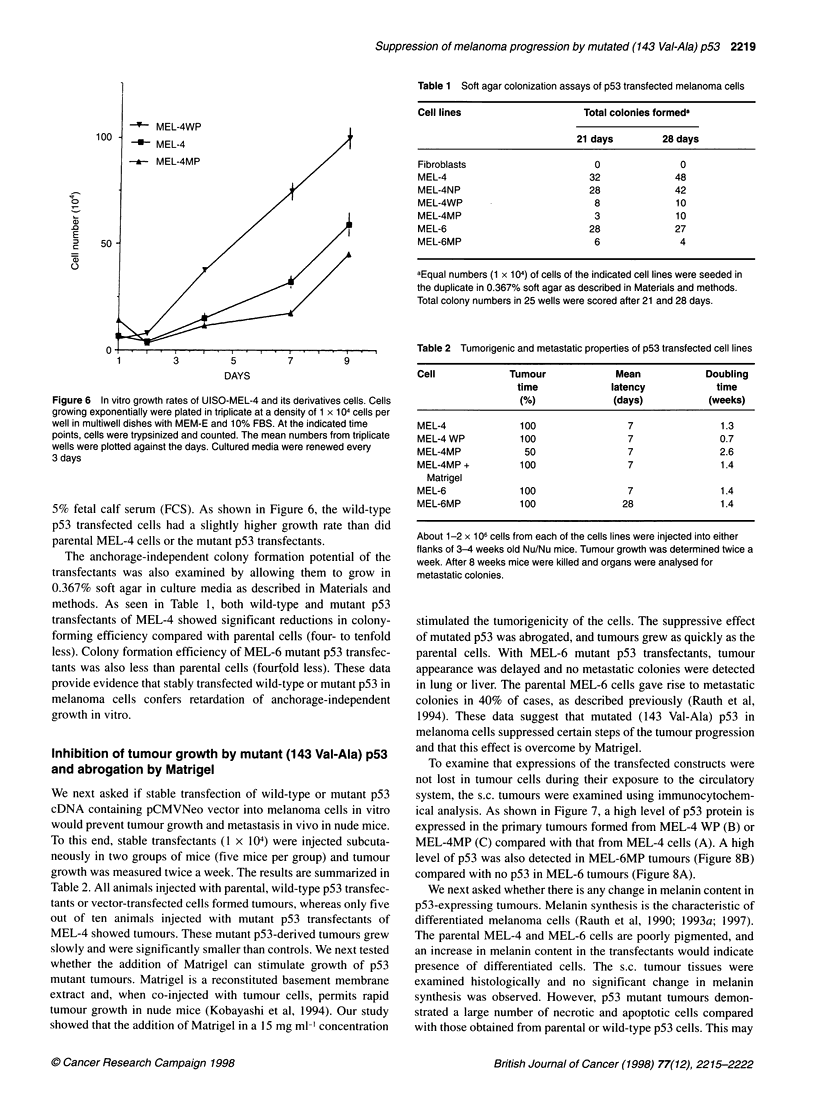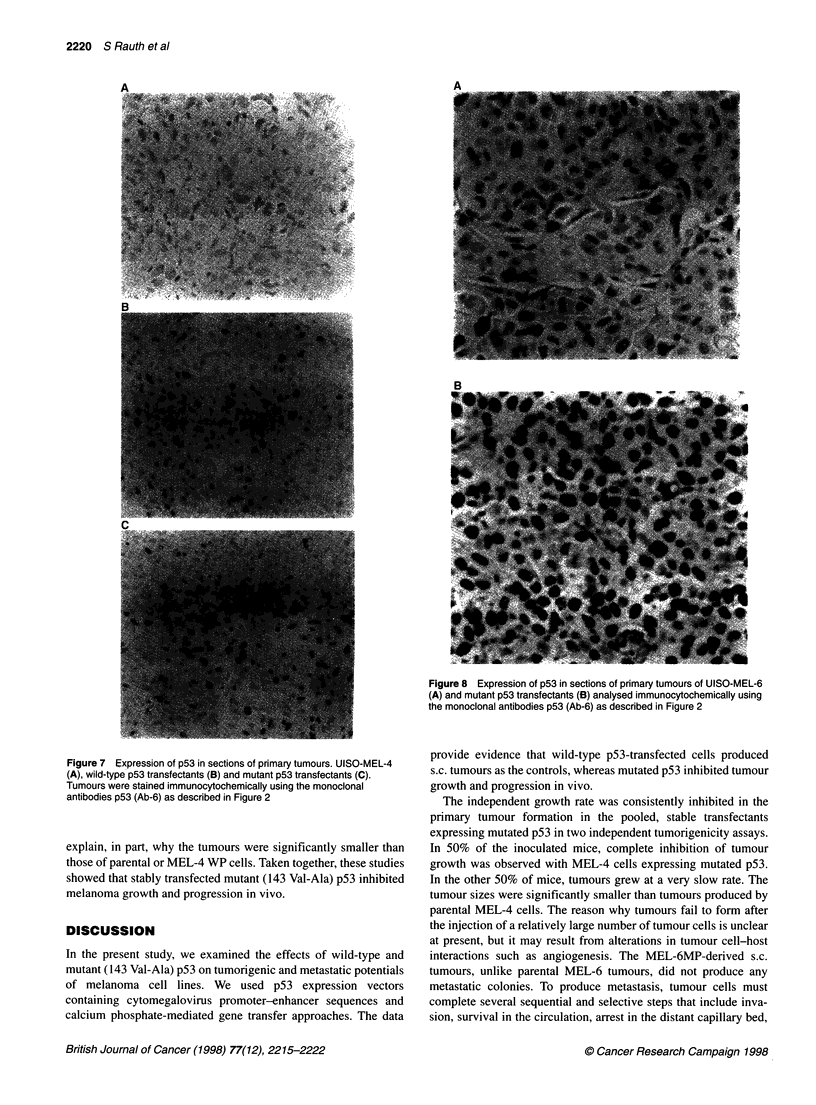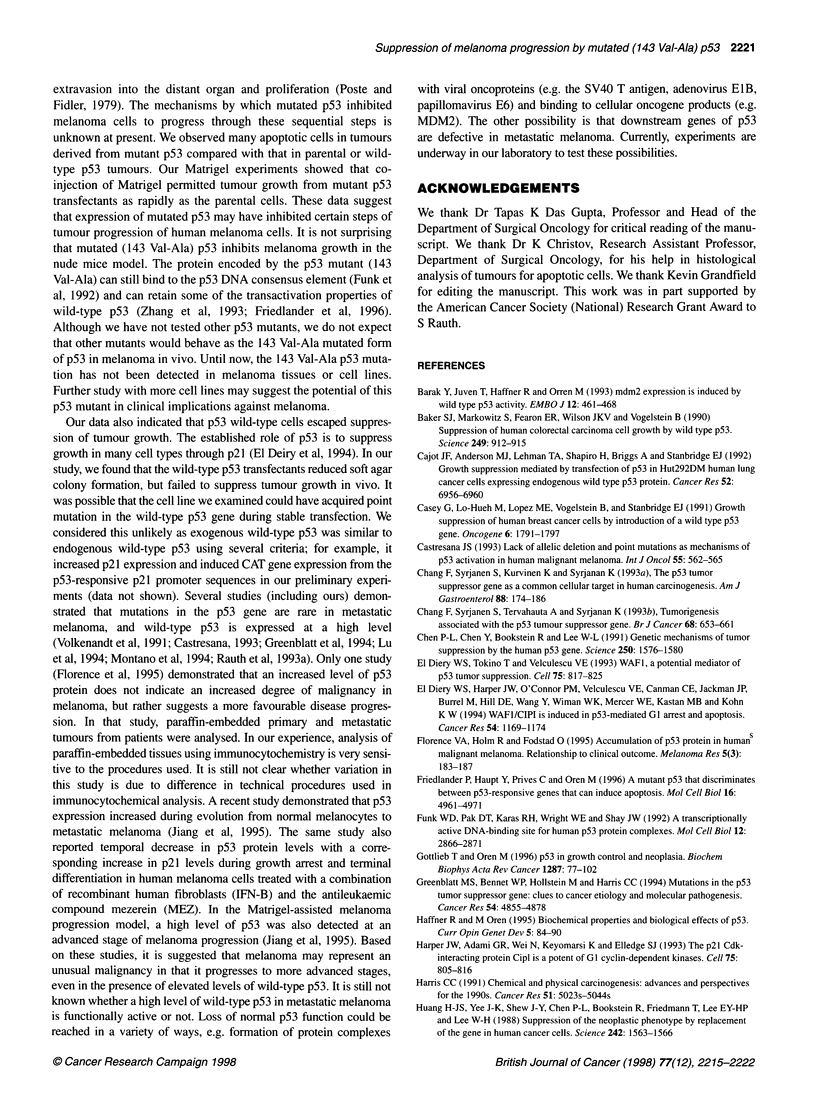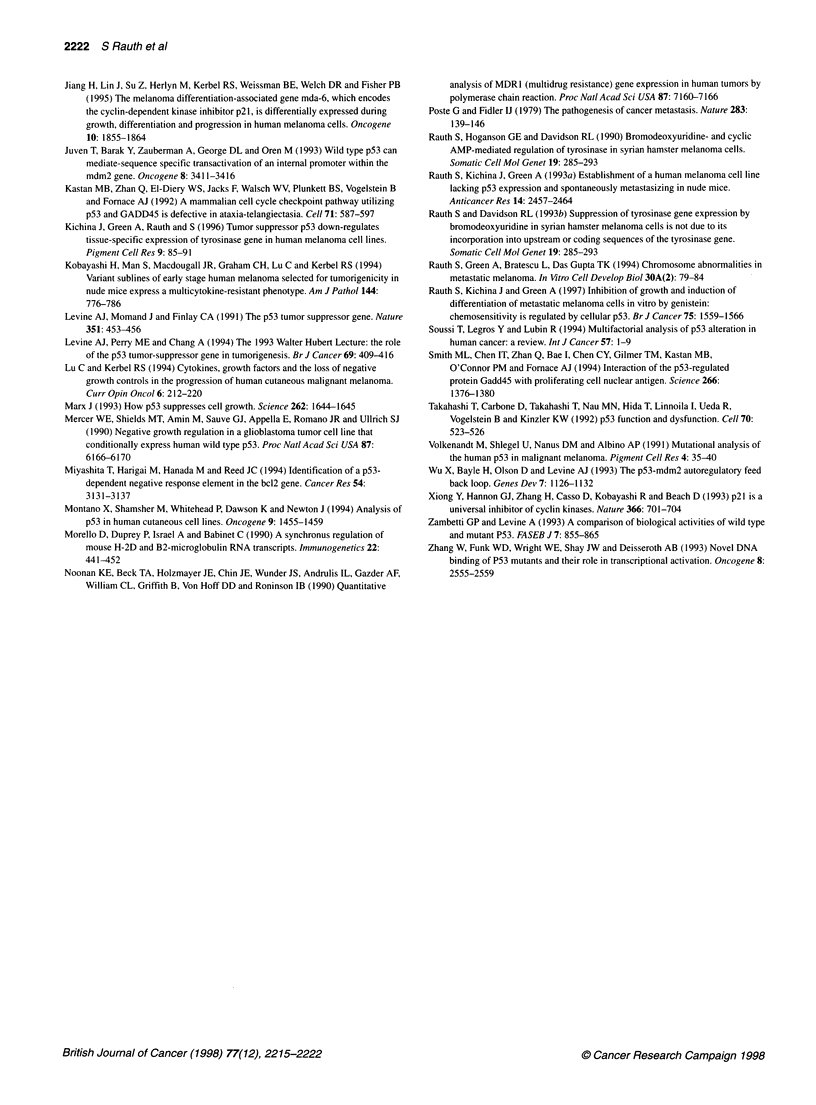# Suppression of tumorigenic and metastatic potentials of human melanoma cell lines by mutated (143 Val-Ala) p53.

**DOI:** 10.1038/bjc.1998.369

**Published:** 1998-06

**Authors:** S. Rauth, A. Green, J. Kichina, A. Shilkaitis

**Affiliations:** Department of Surgical Oncology, University of Illinois at Chicago, 60612, USA.

## Abstract

**Images:**


British Journal of Cancer (1998) 77(12), 2215–2222
© 1998 Cancer Research Campaign

### Suppression of tumorigenic and metastatic potentials of human melanoma cell lines by mutated (143 Val-Ala) p53

**S Rauth[1,2], A Green[1], J Kichina[1,2] and A Shilkaitis[1]**

Departments of [1]Surgical Oncology and [2]Genetics, University of Illinois at Chicago, Chicago, IL 60612, USA

**Summary** Metastatic melanoma, compared with other cancers, appears to be unusual because of its low frequency of p53 mutations and prevalence of wild-type p53 protein in advanced malignancy. Here, we examined the effects of wild-type and mutated p53 (143 Val-Ala) on tumorigenic and metastatic potential of two human melanoma cell lines. The cell line UISO-MEL-4 contains wild-type p53 and is tumorigenic, whereas UISO-MEL-6 lacks p53 and produces lung and liver metastasis upon s.c. injection into athymic mice. Our study showed that UISO-MEL-4 stably transfected with wild-type p53 cDNA driven by cytomegalovirus promoter–enhancer sequences expressed high levels of p53 and p21 and formed s.c. tumours in vivo. Mutated p53 (143 Val-Ala) expression, on the other hand, inhibited tumour growth in 50% of cases and produced significantly slower growing non-metastatic tumours. Reduced tumour growth involved necrotic as well as apoptotic cell death. Inhibition of tumour growth was abrogated by the addition of Matrigel (15 mg $ml^{-1}$). With UISO-MEL-6 cells, stably transfected with mutant p53, tumour growth was delayed and metastasis was inhibited. In soft agar colony formation assay, both wild-type and mutant p53 transfectants reduced anchorage-independent colony formation in vitro. These data suggest that mutated (143 Val-Ala) p53, which retains DNA binding and some of the transactivation functions of the wild-type p53 protein, suppresses tumorigenic and metastatic potentials of human melanoma cell lines in vivo.

**Keywords:** melanoma; p53; tumour growth; metastasis

The tumour-suppressor p53 gene has been found to be mutated in most human malignancies, including tumours of the colon, lung, breast and liver (Harris, 1991; Levine et al, 1991, 1994; Chang et al, 1993*a*, *b*; Soussie et al, 1994). Many of these tumour cells are growth arrested when transfected with the wild-type p53 gene (Baker et al, 1990; Mercer et al, 1990; Casey et al, 1991; Cajot et al, 1992; Takahashi et al, 1992). The effects of wild-type p53 protein on cell growth include cell cycle arrest at $G_1$/S or $G_2$/M check points and cell death through apoptosis (Chen et al, 1991; Marx, 1993; Haffner and Oren, 1995; Gottlieb and Oren, 1996). The growth regulatory functions appeared to be mediated by the interaction of p53 protein with specific DNA sequences, which may allow regulation of transcription of a set of genes involved in growth and apoptotic cell death (Kastan et al, 1992; Wu et al, 1993; Miyashita et al, 1994; Smith et al, 1994). Important genes that are direct targets of p53 include p21/Waf1, which encodes a potent inhibitor of the cyclin-dependent kinases (El Diery et al, 1993, 1994; Harper et al, 1993; Xiong et al, 1993), the growth arrest and DNA damage-inducible gene, GADD45 (Kastan et al, 1992; Smith et al, 1994), and the MDM2 gene, which antagonizes the activity of p53 (Barak et al, 1993; Juven et al, 1993). The DNA binding and transcriptional activity of p53 protein are lost upon specific mutations in the gene (Zambetti and Levine, 1993).

*Received 3 June 1997*
*Revised 21 October 1997*
*Accepted 28 October 1997*

*Correspondence to:* S Rauth, Department of Surgical Oncology (M/C 820), 840 South Wood Street, Chicago, IL 60612, USA

Metastatic melanoma, compared with other cancers, appears to be unusual because of its low frequency of p53 mutations and prevalence of wild-type p53 protein in advanced malignancy (Volkenandt et al, 1991; Castresana, 1993; Greenblatt et al, 1994; Lu and Kerbel, 1994; Montano et al, 1994; Rauth et al, 1993*a*). Several studies, including ours, have reported a much higher level of p53 (2- to 20-fold), mostly wild type, in metastatic melanoma compared with normal melanocytes (Rauth et al, 1993*a*; Jiang et al, 1995). A high level of wild-type p53 has also been detected at the advanced stage of melanoma in Matrigel-assisted melanoma progression model in athymic mice (Jiang et al, 1995). Here, we examined the effects of wild-type and mutated (143 Val-Ala) p53 on tumorigenic and metastatic potentials of two human melanoma cell lines. The cell line UISO-MEL-4 contains wild-type p53 and is tumorigenic, whereas UISO-MEL-6 lacks p53 and is metastatic upon s.c. injection into athymic mice (Rauth et al, 1993*a*, 1994, 1997). We report that UISO-MEL-4 melanoma cells, stably transfected with wild-type p53 expression vector, expressed high levels of p53 and p21 and produced s.c. tumours in vivo as the vector-transfected or parental cells. Mutated p53 (143 Val-Ala) transfectants expressing very high level of p53 protein, on the other hand, inhibited tumour growth in 50% of cases and produced non-metastatic tumours in the rest. Inhibition of tumour growth involved necrotic as well as apoptotic cell death. Inhibition of tumour growth was abrogated by the addition of Matrigel. These data suggest that the protein encoded by the mutant (143 Val-Ala) p53 gene, which retains DNA binding and some of the transcriptional regulatory properties of wild-type p53 (Zhang et al, 1993; Friedlander et al, 1996), suppresses tumorigenic and metastatic potentials of human melanoma cell lines in vivo.

### MATERIALS AND METHODS

### Cells and media

The human melanoma cell lines UISO-MEL-4, UISO-MEL-6 are derived from biopsies obtained from patients with metastatic melanoma (Rauth et al, 1994). Cells were maintained in MEM-H [minimum essential medium with Hanks' balanced salt solution (Gibco, Grand Island, NY, USA)] containing fetal calf serum (2%), L-glutamine (1%), non-essential amino acids and penicillin–streptomycin (0.2%).

### Plasmids and transfection experiments

The human wild-type p53 expression plasmid pC53-SN3, mutant p53 plasmid pC53-SCX3 and the expression vector pCMVNeoBam without p53 cDNA, originally constructed by Bert Vogelstein (Baker et al, 1990), were used in our studies. The p53 expression plasmids contain wild-type or mutant (143 Val-Ala) p53 cDNA sequences under the control of cytomegalovirus (CMV) promoter–enhancer sequences. To isolate cells stably transfected with p53 expression plasmids or the expression vector, UISO-MEL-4 cells were co-transfected with plasmids pC53-SN3 or pC53-SCX3 and PIRVGalNeo as described previously (Rauth et al, 1997). Three days after transfection, the cells were subcultured in the presence of 1 mg $ml^{-1}$ G418 (Sigma Chemical). The selective media with G418 was replaced every 3 days, and colonies that appeared after 10–14 days were pooled and subcultured for further analysis.

### Western blot analysis

For Western blot analysis, transfected cells were lysed in lysis buffer containing 50 mM Tris (pH 8.0), 250 mM sodium chloride and 0.1% Nonidet p-40 as described previously (Rauth et al, 1997). Cell extracts containing equal amounts of protein (150 μg) were separated on a SDS 12.5% polyacrylamide gel. Proteins were transferred electrophoretically to a nitrocellular membrane, and the p53 proteins in the blot were detected with a 1:200 dilution of p53 monoclonal antibody Ab-2 (pAb1801, Oncogene Science) and the enhanced chemiluminescence system (Amersham).

**Figure 1** Western blot analysis for p53 protein expression in UISO-MEL-4 (**A**) and UISO-MEL-6 (**B**) melanoma cells stably transfected with wild-type or mutant p53. The stable transfectants with wild-type p53 were designated as MEL-4WP and had the mutants MEL-4MP or MEL-6MP. p53 protein level was analysed using p53 monoclonal antibody Ab-2 (PAb1801) from Oncogene Science. Cell extracts, each containing 150 μg of total protein, were separated by a SDS 12.5% polyacrylamide gel. p53 protein is detected with 1:200 dilution of p53 monoclonal antibody and chemiluminescence system (Amersham)

### Reverse transcriptase polymerase chain reaction (RT-PCR) for p53 and p21

p53 and p21 mRNAs in the transfected cells were analysed using RT-PCR of total cellular RNA as described previously (Rauth et al, 1993*a*; 1997). To avoid the plateau effect, PCR reaction conditions were optimized and run for 25 cycles. The sequences of the primers used to amplify p53 cDNA are spaced 1225 bp apart and yield full-length cDNA fragment. Primer 1: 5′-AGACTGCCTTC-CGGGTCACT-3′; primer 2: 5′-GGGAACAAAGAAGTGGA-GAAT-3′ as described previously (Kichina et al, 1996). The sequences of the primers used to amplify p21 cDNA were obtained from published sequences (Volkenandt et al, 1991). Primer 1: 5′-GGATGAGTTGGGAGGAGGCA-3′; primer 2: 5′-TTGGAGAAGATCATCCGGCG-3′. These primers yield a 224-bp PCR product. $\beta_2$-Microglobulin ($\beta_2$-m) mRNA, used as an internal control, was also analysed by RT-PCR. The sequences of the primers used to amplify $\beta_2$-microglobulin were obtained from published sequences (Noonan et al, 1990) and yield a 120-bp cDNA fragment.

### Soft agar colony formation assay

Soft agar colony formation assays were performed essentially as described by Huang et al (1988). An equal number of cells ($1 \times 10^4$) from each of the transfectants and parental cells were seeded in 0.367% agar. After 21 and 28 days of incubation at 37°C, the number of colonies was counted.

### Assay for tumorigenicity and metastasis

Melanoma cells were harvested from tissue culture and resuspended in Hanks' balanced salt solution. A total of $1–2 \times 10^6$ viable cells in a 0.2-ml volume were injected subcutaneously in either flank in sets of five mice for each cell line as described (Rauth et al, 1993a). Three- to four-week-old Nu/Nu mice, used in our experiments, were obtained from NCI, Frederick, MD, USA. Tumour growth was determined twice a week by measuring all three diameters [length (*L*), width (*W*) and thickness (*T*)] of the subcutaneous tumour with a vernier caliper. The tumour volume (expressed in $cm^3$) was calculated based on the formula length × width × thickness/6 and by weighing the fresh tumour at the time of autopsy. When the primary tumours reached 2 cm in diameter, mice were killed by carbon dioxide inhalation, and their lungs, livers, kidneys and hearts were removed and examined for the presence of gross and microscopic metastasis. The presence of metastatic melanoma in these tissues was histologically confirmed.

### Histological and immunocytochemical analysis

For histology, primary and metastatic tumour tissues were fixed in 10% formalin, washed in phosphate-buffered saline (PBS), dehydrated in ethanol, and then embedded in paraffin. Sections were

© Cancer Research Campaign 1998

A
B
C

**Figure 2** Immunocytochemical staining of p53 protein in UISO-MEL-4 (**A**) stably transfected with wild-type (**B**) and mutated p53 (**C**). Cells grown on coverslips were analysed for p53 protein using the labelled streptavidin–biotin (LSAB) staining kit (Oncogene Science) and antibody p53 (Ab-6) (Oncogene Science) specific for human wild-type and mutant p53. MAb p3, a non-specific IgGK prepared from BALB/C mouse myeloma p3-X63-88, was used to monitor non-specific binding in the analysis

A
B

**Figure 3** Immunostaining of p53 protein in UISO-MEL-6 (**A**) and its mutant p53 transfectants (**B**). Cells were grown and analysed for p53 protein as described in Figure 2

stained with haematoxylin and eosin. Immunocytochemical analysis of the cells was performed using the streptavidin biotin (LSAB) technique, based on the indirect immunoperoxidase method as described previously (Rauth et al, 1993*a*). The LSAB kit was purchased from Dako, CA, USA, and antibody p53 (Ab-6), specific for human wild-type and mutant p53 was purchased from Oncogene Science. MAb p3, a non-specific IgGk prepared from BALB/c mouse myeloma p3-X63-88 (Rauth et al, 1993*b*), was used to monitor non-specific binding in the analysis. Briefly, cells grown on coverslips were treated with 3% hydrogen peroxide, to inhibit endogenous peroxidase activity, and then with blocking serum (non-immune goat serum). Cells were then incubated with primary antibody, p53 Ab-6, after removing the blocking serum. The link antibody (biotynylated anti-mouse and anti-rabbit immunoglobulin) was then overlaid, followed by PBS wash. Cells were next exposed to streptavidin conjugated to peroxidase, followed by hydrogen peroxide and AEC (3-amino-9-ethylcarbazole, Dako) additions and counter staining with haematoxylin.

### RESULTS

### Expression of wild-type or mutant (143 Val-Ala) p53 genes in UISO MEL-4 and UISO-MEL-6 human melanoma cell lines

To examine the effects of p53 on tumorigenic and metastatic properties of melanoma cells, we chose to transfect two human melanoma cell lines (UISO-MEL-6 and UISO-MEL-4) that either lack or contain wild-type p53. Using the calcium phosphate

© Cancer Research Campaign 1998

**Figure 4** Expression of p53 mRNA in UISO-MEL-4 stable transfectants. p53 mRNA was analysed by reverse transcriptase polymerase chain reaction (RT-PCR). The sequences of the primers used to amplify p53cDNA were spaced 1225 bp apart spanning the entire coding region of the gene. $\beta_2$-microglobulin mRNA, used as an internal control, was also analysed by RT-PCR using primers that yield a 120-bp product. A 10-μl RT-PCR mix was loaded on 2.5% agarose gel

**Figure 5** Expression of p21 mRNA in UISO-MEL-4 human melanoma cells stably transfected with wild-type and mutant p53. p21 mRNA was analysed by reverse transcription and polymerase chain reaction (RT-PCR) using primers that give rise to 224-bp p21 cDNA fragment. $\beta_2$-microglobulin mRNA, used as an internal control, was also analysed by RT-PCR using primers that yield a 120-bp product precipitation procedure, cells were transfected with wild-type or mutant (143 Val-Ala) p53 cDNA containing expression vector, as described previously (Rauth et al, 1997). The (143 Val-Ala) p53 mutant has been shown to retain DNA binding and some of the transactivation function of wild-type p53 (Zhang et al, 1993; Friedlander et al, 1996). As a control, the expression vector pCMVNeoBam without p53 insert was also transfected into the cells under the same conditions. Three days after transfection, the cells were subcultured in the presence of 1 mg $ml^{-1}$ G418. The colonies that appeared after 10–14 days were pooled and expanded so that numerous colonies could be assessed simultaneously. Consistent with the premise that overexpression of wild-type p53 results in a selective disadvantage compared with mutant p53, we observed a lower number of colonies with wild-type p53 compared with that obtained with mutated p53 in the case of MEL-4 cells. In the case of UISO-MEL-6, which is p53 negative, none of the wild-type p53 transfectants survived. The transfectants with wild-type p53 were designated as MEL-4 WP and had the mutants MEL-4 MP or MEL-6MP. The transfectants with expression vector were designated as MEL-4 NP or MEL-6NP

The stably transfected cells were then analysed for p53 protein expression using Western blot hybridization with p53 monoclonal antibody Ab-2 (PAb1801, Oncogene Science). As shown in Figure 1A, high levels of p53 proteins were detected in MEL-4 WP and MEL-4 MP compared with parental MEL-4 cells. The MEL-4 MP cells expressed a very high level of p53 protein, and this accumulation could be due to stabilization of the mutant p53 by complex formation with wild-type p53 or other proteins (Zambetti et al, 1993). Figure 1B shows a high level of p53 protein in MEL-6MP and no p53 in parental MEL-6 cell line. To examine whether the transfectants express p53 uniformly in level and localization, p53 expression was also analysed immunocytochemically using p53 monoclonal antibody Ab-6 (Oncogene Science). As shown in Figure 2, a high level of uniform immunostaining, both nuclear and cytoplasmic, was detected in p53 transfectants of the MEL-4 cell line. Figure 3 shows high level of uniform immunostaining in MEL-6MP cells.

To determine whether a high level of p53 expression is not due to accumulation of the protein but due to transcription from the transfected plasmids, we analysed total cellular RNA from the transfectants using RT-PCR. As shown in Figure 4, MEL-4 WP or MP expressed a higher level of p53 transcript than parental MEL-4 cells. As an internal control for p53 expression, we analysed $\beta_2$-m mRNA levels using RT-PCR. The $\beta_2$-m gene is ubiquitously expressed (Morrelo et al, 1990) and is used to monitor the levels of cDNA in RT-PCR. The $\beta_2$-m band appears to be similar in intensity for all cell types and therefore indicates equal RNA loading of all samples. Taken together, these results show that p53 was expressed from the transfected plasmids in human melanoma cells.

Expression of the p21 gene is directly induced by wild-type p53 and is an important mediator of p53-dependent tumour growth suppression (El Diery et al, 1994). To determine whether p53 in the stable transfectants induced p21 gene expression, p21 mRNA expression was analysed using RT-PCR. As shown in Figure 5, an increased level of p21 mRNA was detected in the wild-type p53 transfectants. $\beta_2$-Microglobulin mRNA, analysed using RT-PCR, remained unchanged in both wild-type and mutant p53 transfectants. The levels of p21 protein in the stable transfectants were also analysed by immunocytochemical analysis using antibody Waf1 (Ab-1). A higher level of immunostaining was detected in p53 wild-type transfectants compared with parental MEL-4 (data not shown), indicating the presence of high levels of p21 protein. These results show that wild-type p53 expressed from the transfected plasmid induced p21 expression in MEL-4 WP cells.

### *In vitro* growth characteristics of p53 transfectants

The anchorage-dependent growth rates of wild-type and mutant p53 transfectants were then examined in culture media containing

*© Cancer Research Campaign 1998*

**Figure 6** In vitro growth rates of UISO-MEL-4 and its derivatives cells. Cells growing exponentially were plated in triplicate at a density of 1 × $10^4$ cells per well in multiwell dishes with MEM-E and 10% FBS. At the indicated time points, cells were trypsinized and counted. The mean numbers from triplicate wells were plotted against the days. Cultured media were renewed every 3 days

5% fetal calf serum (FCS). As shown in Figure 6, the wild-type p53 transfected cells had a slightly higher growth rate than did parental MEL-4 cells or the mutant p53 transfectants.

The anchorage-independent colony formation potential of the transfectants was also examined by allowing them to grow in 0.367% soft agar in culture media as described in Materials and methods. As seen in Table 1, both wild-type and mutant p53 transfectants of MEL-4 showed significant reductions in colony-forming efficiency compared with parental cells (four- to tenfold less). Colony formation efficiency of MEL-6 mutant p53 transfectants was also less than parental cells (fourfold less). These data provide evidence that stably transfected wild-type or mutant p53 in melanoma cells confers retardation of anchorage-independent growth in vitro.

### Inhibition of tumour growth by mutant (143 Val-Ala) p53 and abrogation by Matrigel

We next asked if stable transfection of wild-type or mutant p53 cDNA containing pCMVNeo vector into melanoma cells in vitro would prevent tumour growth and metastasis in vivo in nude mice. To this end, stable transfectants (1 × $10^4$) were injected subcutaneously in two groups of mice (five mice per group) and tumour growth was measured twice a week. The results are summarized in Table 2. All animals injected with parental, wild-type p53 transfectants or vector-transfected cells formed tumours, whereas only five out of ten animals injected with mutant p53 transfectants of MEL-4 showed tumours. These mutant p53-derived tumours grew slowly and were significantly smaller than controls. We next tested whether the addition of Matrigel can stimulate growth of p53 mutant tumours. Matrigel is a reconstituted basement membrane extract and, when co-injected with tumour cells, permits rapid tumour growth in nude mice (Kobayashi et al, 1994). Our study showed that the addition of Matrigel in a 15 mg $ml^{-1}$ concentration

**Table 1** Soft agar colonization assays of p53 transfected melanoma cells

| Cell lines | Total colonies formed[a] | |
|---|---|---|
| | 21 days | 28 days |
| Fibroblasts | 0 | 0 |
| MEL-4 | 32 | 48 |
| MEL-4NP | 28 | 42 |
| MEL-4WP | 8 | 10 |
| MEL-4MP | 3 | 10 |
| MEL-6 | 28 | 27 |
| MEL-6MP | 6 | 4 |

[a]Equal numbers (1 × $10^4$) of cells of the indicated cell lines were seeded in the duplicate in 0.367% soft agar as described in Materials and methods. Total colony numbers in 25 wells were scored after 21 and 28 days.

**Table 2** Tumorigenic and metastatic properties of p53 transfected cell lines

| Cell | Tumour time (%) | Mean latency (days) | Doubling time (weeks) |
|---|---|---|---|
| MEL-4 | 100 | 7 | 1.3 |
| MEL-4 WP | 100 | 7 | 0.7 |
| MEL-4MP | 50 | 7 | 2.6 |
| MEL-4MP + Matrigel | 100 | 7 | 1.4 |
| MEL-6 | 100 | 7 | 1.4 |
| MEL-6MP | 100 | 28 | 1.4 |

About 1–2 × $10^6$ cells from each of the cells lines were injected into either flanks of 3–4 weeks old Nu/Nu mice. Tumour growth was determined twice a week. After 8 weeks mice were killed and organs were analysed for metastatic colonies.

stimulated the tumorigenicity of the cells. The suppressive effect of mutated p53 was abrogated, and tumours grew as quickly as the parental cells. With MEL-6 mutant p53 transfectants, tumour appearance was delayed and no metastatic colonies were detected in lung or liver. The parental MEL-6 cells gave rise to metastatic colonies in 40% of cases, as described previously (Rauth et al, 1994). These data suggest that mutated (143 Val-Ala) p53 in melanoma cells suppressed certain steps of the tumour progression and that this effect is overcome by Matrigel.

To examine that expressions of the transfected constructs were not lost in tumour cells during their exposure to the circulatory system, the s.c. tumours were examined using immunocytochemical analysis. As shown in Figure 7, a high level of p53 protein is expressed in the primary tumours formed from MEL-4 WP (B) or MEL-4MP (C) compared with that from MEL-4 cells (A). A high level of p53 was also detected in MEL-6MP tumours (Figure 8B) compared with no p53 in MEL-6 tumours (Figure 8A).

We next asked whether there is any change in melanin content in p53-expressing tumours. Melanin synthesis is the characteristic of differentiated melanoma cells (Rauth et al, 1990; 1993*a*; 1997). The parental MEL-4 and MEL-6 cells are poorly pigmented, and an increase in melanin content in the transfectants would indicate presence of differentiated cells. The s.c. tumour tissues were examined histologically and no significant change in melanin synthesis was observed. However, p53 mutant tumours demonstrated a large number of necrotic and apoptotic cells compared with those obtained from parental or wild-type p53 cells. This may

© Cancer Research Campaign 1998

**Figure 7** Expression of p53 in sections of primary tumours. UISO-MEL-4 (**A**), wild-type p53 transfectants (**B**) and mutant p53 transfectants (**C**). Tumours were stained immunocytochemically using the monoclonal antibodies p53 (Ab-6) as described in Figure 2

explain, in part, why the tumours were significantly smaller than those of parental or MEL-4 WP cells. Taken together, these studies showed that stably transfected mutant (143 Val-Ala) p53 inhibited melanoma growth and progression in vivo.

### DISCUSSION

In the present study, we examined the effects of wild-type and mutant (143 Val-Ala) p53 on tumorigenic and metastatic potentials of melanoma cell lines. We used p53 expression vectors containing cytomegalovirus promoter–enhancer sequences and calcium phosphate-mediated gene transfer approaches. The data provide evidence that wild-type p53-transfected cells produced s.c. tumours as the controls, whereas mutated p53 inhibited tumour growth and progression in vivo.

**Figure 8** Expression of p53 in sections of primary tumours of UISO-MEL-6 (**A**) and mutant p53 transfectants (**B**) analysed immunocytochemically using the monoclonal antibodies p53 (Ab-6) as described in Figure 2

The independent growth rate was consistently inhibited in the primary tumour formation in the pooled, stable transfectants expressing mutated p53 in two independent tumorigenicity assays. In 50% of the inoculated mice, complete inhibition of tumour growth was observed with MEL-4 cells expressing mutated p53. In the other 50% of mice, tumours grew at a very slow rate. The tumour sizes were significantly smaller than tumours produced by parental MEL-4 cells. The reason why tumours fail to form after the injection of a relatively large number of tumour cells is unclear at present, but it may result from alterations in tumour cell–host interactions such as angiogenesis. The MEL-6MP-derived s.c. tumours, unlike parental MEL-6 tumours, did not produce any metastatic colonies. To produce metastasis, tumour cells must complete several sequential and selective steps that include invasion, survival in the circulation, arrest in the distant capillary bed,

 © Cancer Research Campaign 1998

extravasion into the distant organ and proliferation (Poste and Fidler, 1979). The mechanisms by which mutated p53 inhibited melanoma cells to progress through these sequential steps is unknown at present. We observed many apoptotic cells in tumours derived from mutant p53 compared with that in parental or wild-type p53 tumours. Our Matrigel experiments showed that co-injection of Matrigel permitted tumour growth from mutant p53 transfectants as rapidly as the parental cells. These data suggest that expression of mutated p53 may have inhibited certain steps of tumour progression of human melanoma cells. It is not surprising that mutated (143 Val-Ala) p53 inhibits melanoma growth in the nude mice model. The protein encoded by the p53 mutant (143 Val-Ala) can still bind to the p53 DNA consensus element (Funk et al, 1992) and can retain some of the transactivation properties of wild-type p53 (Zhang et al, 1993; Friedlander et al, 1996). Although we have not tested other p53 mutants, we do not expect that other mutants would behave as the 143 Val-Ala mutated form of p53 in melanoma in vivo. Until now, the 143 Val-Ala p53 mutation has not been detected in melanoma tissues or cell lines. Further study with more cell lines may suggest the potential of this p53 mutant in clinical implications against melanoma.

Our data also indicated that p53 wild-type cells escaped suppression of tumour growth. The established role of p53 is to suppress growth in many cell types through p21 (El Deiry et al, 1994). In our study, we found that the wild-type p53 transfectants reduced soft agar colony formation, but failed to suppress tumour growth in vivo. It was possible that the cell line we examined could have acquired point mutation in the wild-type p53 gene during stable transfection. We considered this unlikely as exogenous wild-type p53 was similar to endogenous wild-type p53 using several criteria; for example, it increased p21 expression and induced CAT gene expression from the p53-responsive p21 promoter sequences in our preliminary experiments (data not shown). Several studies (including ours) demonstrated that mutations in the p53 gene are rare in metastatic melanoma, and wild-type p53 is expressed at a high level (Volkenandt et al, 1991; Castresana, 1993; Greenblatt et al, 1994; Lu et al, 1994; Montano et al, 1994; Rauth et al, 1993a). Only one study (Florence et al, 1995) demonstrated that an increased level of p53 protein does not indicate an increased degree of malignancy in melanoma, but rather suggests a more favourable disease progression. In that study, paraffin-embedded primary and metastatic tumours from patients were analysed. In our experience, analysis of paraffin-embedded tissues using immunocytochemistry is very sensitive to the procedures used. It is still not clear whether variation in this study is due to difference in technical procedures used in immunocytochemical analysis. A recent study demonstrated that p53 expression increased during evolution from normal melanocytes to metastatic melanoma (Jiang et al, 1995). The same study also reported temporal decrease in p53 protein levels with a corresponding increase in p21 levels during growth arrest and terminal differentiation in human melanoma cells treated with a combination of recombinant human fibroblasts (IFN-B) and the antileukaemic compound mezerein (MEZ). In the Matrigel-assisted melanoma progression model, a high level of p53 was also detected at an advanced stage of melanoma progression (Jiang et al, 1995). Based on these studies, it is suggested that melanoma may represent an unusual malignancy in that it progresses to more advanced stages, even in the presence of elevated levels of wild-type p53. It is still not known whether a high level of wild-type p53 in metastatic melanoma is functionally active or not. Loss of normal p53 function could be reached in a variety of ways, e.g. formation of protein complexes with viral oncoproteins (e.g. the SV40 T antigen, adenovirus E1B, papillomavirus E6) and binding to cellular oncogene products (e.g. MDM2). The other possibility is that downstream genes of p53 are defective in metastatic melanoma. Currently, experiments are underway in our laboratory to test these possibilities.

### ACKNOWLEDGEMENTS

We thank Dr Tapas K Das Gupta, Professor and Head of the Department of Surgical Oncology for critical reading of the manuscript. We thank Dr K Christov, Research Assistant Professor, Department of Surgical Oncology, for his help in histological analysis of tumours for apoptotic cells. We thank Kevin Grandfield for editing the manuscript. This work was in part supported by the American Cancer Society (National) Research Grant Award to S Rauth.

### REFERENCES

Barak Y, Juven T, Haffner R and Orren M (1993) mdm2 expression is induced by wild type p53 activity. *EMBO J* **12**: 461–468

Baker SJ, Markowitz S, Fearon ER, Wilson JKV and Vogelstein B (1990) Suppression of human colorectal carcinoma cell growth by wild type p53. *Science* **249**: 912–915

Cajot JF, Anderson MJ, Lehman TA, Shapiro H, Briggs A and Stanbridge EJ (1992) Growth suppression mediated by transfection of p53 in Hut292DM human lung cancer cells expressing endogenous wild type p53 protein. *Cancer Res* **52**: 6956–6960

Casey G, Lo-Hueh M, Lopez ME, Vogelstein B, and Stanbridge EJ (1991) Growth suppression of human breast cancer cells by introduction of a wild type p53 gene. *Oncogene* **6**: 1791–1797

Castresana JS (1993) Lack of allelic deletion and point mutations as mechanisms of p53 activation in human malignant melanoma. *Int J Oncol* **55**: 562–565

Chang F, Syrjanen S, Kurvinen K and Syrjanan K (1993*a*), The p53 tumor suppressor gene as a common cellular target in human carcinogenesis. *Am J Gastroenterol* **88**: 174–186

Chang F, Syrjanen S, Tervahauta A and Syrjanan K (1993*b*), Tumorigenesis associated with the p53 tumour suppressor gene. *Br J Cancer* **68**: 653–661

Chen P-L, Chen Y, Bookstein R and Lee W-L (1991) Genetic mechanisms of tumor suppression by the human p53 gene. *Science* **250**: 1576–1580

El Diery WS, Tokino T and Velculescu VE (1993) WAF1, a potential mediator of p53 tumor suppression. *Cell* **75**: 817–825

El Diery WS, Harper JW, O'Connor PM, Velculescu VE, Canman CE, Jackman JP, Burrel M, Hill DE, Wang Y, Wiman WK, Mercer WE, Kastan MB and Kohn K W (1994) WAF1/CIPI is induced in p53-mediated G1 arrest and apoptosis. *Cancer Res* **54**: 1169–1174

Florence VA, Holm R and Fodstad O (1995) Accumulation of p53 protein in human[S] malignant melanoma. Relationship to clinical outcome. *Melanoma Res* **5(3)**: 183–187

Friedlander P, Haupt Y, Prives C and Oren M (1996) A mutant p53 that discriminates between p53-responsive genes that can induce apoptosis. *Mol Cell Biol* **16**: 4961–4971

Funk WD, Pak DT, Karas RH, Wright WE and Shay JW (1992) A transcriptionally active DNA-binding site for human p53 protein complexes. *Mol Cell Biol* **12**: 2866–2871

Gottlieb T and Oren M (1996) p53 in growth control and neoplasia. *Biochem Biophys Acta Rev Cancer* **1287**: 77–102

Greenblatt MS, Bennet WP, Hollstein M and Harris CC (1994) Mutations in the p53 tumor suppressor gene: clues to cancer etiology and molecular pathogenesis. *Cancer Res* **54**: 4855–4878

Haffner R and M Oren (1995) Biochemical properties and biological effects of p53. *Curr Opin Genet Dev* **5**: 84–90

Harper JW, Adami GR, Wei N, Keyomarsi K and Elledge SJ (1993) The p21 Cdk-interacting protein Cipl is a potent of G1 cyclin-dependent kinases. *Cell* **75**: 805–816

Harris CC (1991) Chemical and physical carcinogenesis: advances and perspectives for the 1990s. *Cancer Res* **51**: 5023s–5044s

Huang H-JS, Yee J-K, Shew J-Y, Chen P-L, Bookstein R, Friedmann T, Lee EY-HP and Lee W-H (1988) Suppression of the neoplastic phenotype by replacement of the gene in human cancer cells. *Science* **242**: 1563–1566

© Cancer Research Campaign 1998 

Jiang H, Lin J, Su Z, Herlyn M, Kerbel RS, Weissman BE, Welch DR and Fisher PB (1995) The melanoma differentiation-associated gene mda-6, which encodes the cyclin-dependent kinase inhibitor p21, is differentially expressed during growth, differentiation and progression in human melanoma cells. *Oncogene* **10**: 1855–1864

Juven T, Barak Y, Zauberman A, George DL and Oren M (1993) Wild type p53 can mediate-sequence specific transactivation of an internal promoter within the mdm2 gene. *Oncogene* **8**: 3411–3416

Kastan MB, Zhan Q, El-Diery WS, Jacks F, Walsch WV, Plunkett BS, Vogelstein B and Fornace AJ (1992) A mammalian cell cycle checkpoint pathway utilizing p53 and GADD45 is defective in ataxia-telangiectasia. *Cell* **71**: 587–597

Kichina J, Green A, Rauth and S (1996) Tumor suppressor p53 down-regulates tissue-specific expression of tyrosinase gene in human melanoma cell lines. *Pigment Cell Res* **9**: 85–91

Kobayashi H, Man S, Macdougall JR, Graham CH, Lu C and Kerbel RS (1994) Variant sublines of early stage human melanoma selected for tumorigenicity in nude mice express a multicytokine-resistant phenotype. *Am J Pathol* **144**: 776–786

Levine AJ, Momand J and Finlay CA (1991) The p53 tumor suppressor gene. *Nature* **351**: 453–456

Levine AJ, Perry ME and Chang A (1994) The 1993 Walter Hubert Lecture: the role of the p53 tumor-suppressor gene in tumorigenesis. *Br J Cancer* **69**: 409–416

Lu C and Kerbel RS (1994) Cytokines, growth factors and the loss of negative growth controls in the progression of human cutaneous malignant melanoma. *Curr Opin Oncol* **6**: 212–220

Marx J (1993) How p53 suppresses cell growth. *Science* **262**: 1644–1645

Mercer WE, Shields MT, Amin M, Sauve GJ, Appella E, Romano JR and Ullrich SJ (1990) Negative growth regulation in a glioblastoma tumor cell line that conditionally express human wild type p53. *Proc Natl Acad Sci USA* **87**: 6166–6170

Miyashita T, Harigai M, Hanada M and Reed JC (1994) Identification of a p53-dependent negative response element in the bcl2 gene. *Cancer Res* **54**: 3131–3137

Montano X, Shamsher M, Whitehead P, Dawson K and Newton J (1994) Analysis of p53 in human cutaneous cell lines. *Oncogene* **9**: 1455–1459

Morello D, Duprey P, Israel A and Babinet C (1990) A synchronus regulation of mouse H-2D and B2-microglobulin RNA transcripts. *Immunogenetics* **22**: 441–452

Noonan KE, Beck TA, Holzmayer JE, Chin JE, Wunder JS, Andrulis IL, Gazder AF, William CL, Griffith B, Von Hoff DD and Roninson IB (1990) Quantitative analysis of MDR1 (multidrug resistance) gene expression in human tumors by polymerase chain reaction. *Proc Natl Acad Sci USA* **87**: 7160–7166

Poste G and Fidler IJ (1979) The pathogenesis of cancer metastasis. *Nature* **283**: 139–146

Rauth S, Hoganson GE and Davidson RL (1990) Bromodeoxyuridine- and cyclic AMP-mediated regulation of tyrosinase in syrian hamster melanoma cells. *Somatic Cell Mol Genet* **19**: 285–293

Rauth S, Kichina J, Green A (1993*a*) Establishment of a human melanoma cell line lacking p53 expression and spontaneously metastasizing in nude mice. *Anticancer Res* **14**: 2457–2464

Rauth S and Davidson RL (1993*b*) Suppression of tyrosinase gene expression by bromodeoxyuridine in syrian hamster melanoma cells is not due to its incorporation into upstream or coding sequences of the tyrosinase gene. *Somatic Cell Mol Genet* **19**: 285–293

Rauth S, Green A, Bratescu L, Das Gupta TK (1994) Chromosome abnormalities in metastatic melanoma. *In Vitro Cell Develop Biol* **30A(2)**: 79–84

Rauth S, Kichina J and Green A (1997) Inhibition of growth and induction of differentiation of metastatic melanoma cells in vitro by genistein: chemosensitivity is regulated by cellular p53. *Br J Cancer* **75**: 1559–1566

Soussi T, Legros Y and Lubin R (1994) Multifactorial analysis of p53 alteration in human cancer: a review. *Int J Cancer* **57**: 1–9

Smith ML, Chen IT, Zhan Q, Bae I, Chen CY, Gilmer TM, Kastan MB, O'Connor PM and Fornace AJ (1994) Interaction of the p53-regulated protein Gadd45 with proliferating cell nuclear antigen. *Science* **266**: 1376–1380

Takahashi T, Carbone D, Takahashi T, Nau MN, Hida T, Linnoila I, Ueda R, Vogelstein B and Kinzler KW (1992) p53 function and dysfunction. *Cell* **70**: 523–526

Volkenandt M, Shlegel U, Nanus DM and Albino AP (1991) Mutational analysis of the human p53 in malignant melanoma. *Pigment Cell Res* **4**: 35–40

Wu X, Bayle H, Olson D and Levine AJ (1993) The p53-mdm2 autoregulatory feed back loop. *Genes Dev* **7**: 1126–1132

Xiong Y, Hannon GJ, Zhang H, Casso D, Kobayashi R and Beach D (1993) p21 is a universal inhibitor of cyclin kinases. *Nature* **366**: 701–704

Zambetti GP and Levine A (1993) A comparison of biological activities of wild type and mutant P53. *FASEB J* **7**: 855–865

Zhang W, Funk WD, Wright WE, Shay JW and Deisseroth AB (1993) Novel DNA binding of P53 mutants and their role in transcriptional activation. *Oncogene* **8**: 2555–2559

© Cancer Research Campaign 1998